# Retinoic acid-induced CYP51 nuclear translocation promotes meiosis prophase I process and is correlated to the expression of REC8 and STAG3 in mice

**DOI:** 10.1242/bio.035626

**Published:** 2018-11-15

**Authors:** Xinyi Mu, Jia Wen, Qian Chen, Zhengpin Wang, Yijing Wang, Meng Guo, Yi Yang, JinRui Xu, Zhiqing Wei, Guoliang Xia, Mengye Yang, Chao Wang

**Affiliations:** 1 State Key Laboratory of Agrobiotechnology, College of Biological Sciences, China Agricultural University, Beijing 100193, China; 2 Department of Histology and Embryology, Chongqing Medical University, Chongqing 400016, China; 3 Department of Laboratory Animal Science, School of Basic Medical Science, Capital Medical University, Beijing 100069, China; 4 Key Laboratory of Ministry of Education for Conservation and Utilization of Special Biological Resources in the Western China, College of Life Science, Ningxia University, 539 W Helanshan Road, Xixia District, Yinchuan, Ningxia 750021, China; 5 Department of Biochemistry, College of Life Sciences, Wuhan University, Luojia Hill, Wuhan 430072, China

**Keywords:** CYP51, Oocytes, Meiosis, Chromosome synapsis, Primordial follicle formation

## Abstract

Lanosterol 14 α-demethylase (CYP51) plays a crucial role in cholesterol biosynthesis. In gamete development, CYP51 is involved in initiating meiosis resumption in oocytes through its product, meiosis activating sterol (MAS). In this study, CYP51 was observed to localize within the nucleus of germ cells undergoing meiotic prophase I. Following the addition of retinoic acid (RA) to induce meiosis or the RA receptor pan-antagonist AGN193109 to block meiosis in fetal ovaries, the translocation of CYP51 into the nucleus of oocytes was advanced or delayed, respectively. In addition, treatment with *Cyp51*-siRNA or RS21745, a specific CYP51 inhibitor, significantly delayed the meiotic progression of oocytes in the ovary, with most oocytes arresting at the zygotene stage, and likewise, significantly reduced perinatal primordial follicle formation. Furthermore, inhibition of CYP51 is correlated to significantly decreased expression of REC8 and STAG3, both of which are meiosis-specific cohesin subunits. To sum up, RA-induced CYP51 nuclear translocation is critical for oocytes meiotic progression, and consequently folliculogenesis, which might act through impacting the expression of meiosis-specific cohesins REC8 and STAG3.

## Abstract (precis)

**Summary:** CYP51 displays cytoplasm-to-nucleus translocation in germ cells in mice. CYP51 participates in germ cell meiotic progression and folliculogenesis via regulating the expression of cohesin REC8 and STAG3.

## INTRODUCTION

Meiosis, which is the basis of sexual reproduction, consists of a series of highly coordinated events resulting in the generation of haploid gametes from a diploid precursor. In female mice gonads, germ cells generally enter meiosis, a process that has been shown to be induced by retinoic acid (RA), at approximately 13.5 days post-coitus (dpc) (Baltus et al., 2006; Bowles et al., 2006; Koubova et al., 2006; Li et al., 2011). Oocytes progress through meiosis prophase I in a stepwise fashion. During the leptotene stage, SPO11-dependent double-strand breaks (DSBs) are introduced in the structure of DNA and homologous recombination begins (Roeder, 1997). In the zygotene stage, homologs begin to synapse at the homologous pairing sites, and the classical tripartite structure of the synaptonemal complex (SC) becomes visible (Vazquez-Nin and Echeverria, 1976). While in the pachytene stage, the homologs are fully synapsed and recombination has completed. Finally, the oocytes enter the diplotene stage, which begins at approximately 17.5 dpc in mice (Borum, 1961), and are then arrested at the late diplotene stage, which is commonly known as the dictyate stage. As far as we know, RA regulates the chromosomal program in germ cells by inducing expressions of various meiotic prophase genes (Soh et al., 2015); however, the downstream regulation mechanism is still intriguing.

The chromosome gymnastics that occur in meiosis are influenced by the cohesin complex, which holds sister chromatids together during the first division and mediates their regulated separation during the second division. During prophase I, cohesin interacts with the SC components and binds to the ‘lateral elements’, which is crucial for proper homolog synapses (Eijpe et al., 2003; Ishiguro et al., 2011; Lee and Hirano, 2011; Lee et al., 2003; Pelttari et al., 2001; Prieto et al., 2001; Revenkova et al., 2001). A recent study showed that *Rec8*, which encodes a key component of cohesin complex, is directly induced by RA, independent of *Stra8* function (Koubova et al., 2014). However, the faithful regulation of cohesin expression and behaviors during meiotic prophase I remain largely unclear.

In female mammals, a fixed population of primordial follicles is established in ovary perinatally, and serves as the whole resource of developing follicles and oocytes throughout reproductive life. Primordial follicle formation is systematically orchestrated by signals from both somatic and germ cells (Wang et al., 2017); one oocyte, which has reached the diplotene stage, interacts with and is surrounded by several pre-granulosa cells to form single primordial follicle. It is suggested that sufficient primordial follicle assembly may depend upon accurate meiotic progression in oocytes (Grive et al., 2014; Wang et al., 2015). Nevertheless, molecular affairs involved in regulating primordial follicle formation are far from being well studied.

Lanosterol 14 α-demethylase (CYP51) is a member of the cytochrome P450 gene superfamily and is the only CYP protein found in both prokaryotes and eukaryotes. As an enzyme that plays a crucial role in cholesterol biosynthesis, CYP51 catalyzes the formation of follicular fluid meiosis-activating sterol (FF-MAS) from lanosterol (Rozman and Waterman, 1998). FF-MAS was suggested to be the ‘meiosis-inducing substance’ secreted by the female fetal gonad that could initiate fetal male germ cell meiosis (Byskov and Saxén, 1976), and was also found to be capable of reinitiating meiotic resumption of mice oocytes at a later stage *in vitro* (Byskov et al., 1995; Grondahl et al., 1998; Wang et al., 2009). We have previously shown that CYP51 is highly expressed in fetal ovaries while germ cells undergo meiotic prophase I and specific inhibition of CYP51 reduced primordial follicle formation (Zhang et al., 2009). Nevertheless, the functions of CYP51 in the early meiotic stage of germ cell development remain unclear.

This study was dedicated to examining the relationship between RA and CYP51 in mouse meiosis prophase I, as well as to elucidating the possible mechanisms by which CYP51 regulates meiosis progression and primordial follicle formation. The results showed that CYP51 was translocated to the nucleus of germ cells during meiotic prophase I following the regulation of RA, and inhibition of CYP51 arrested oocyte meiosis progression at the zygotene stage. In addition, the expressions of REC8 and STAG3 were significantly inhibited by a CYP51 inhibitor, suggesting that CYP51 might participate in homologous chromosome synapsis and its function was correlated with cohesin expression. These results may enable researchers to gain a better understanding of the role of CYP51 in the spatiotemporal regulation of chromosomal progression during meiotic prophase I.

## RESULTS

### Localization of CYP51 in developing mouse gonad

**Fig. 1.**
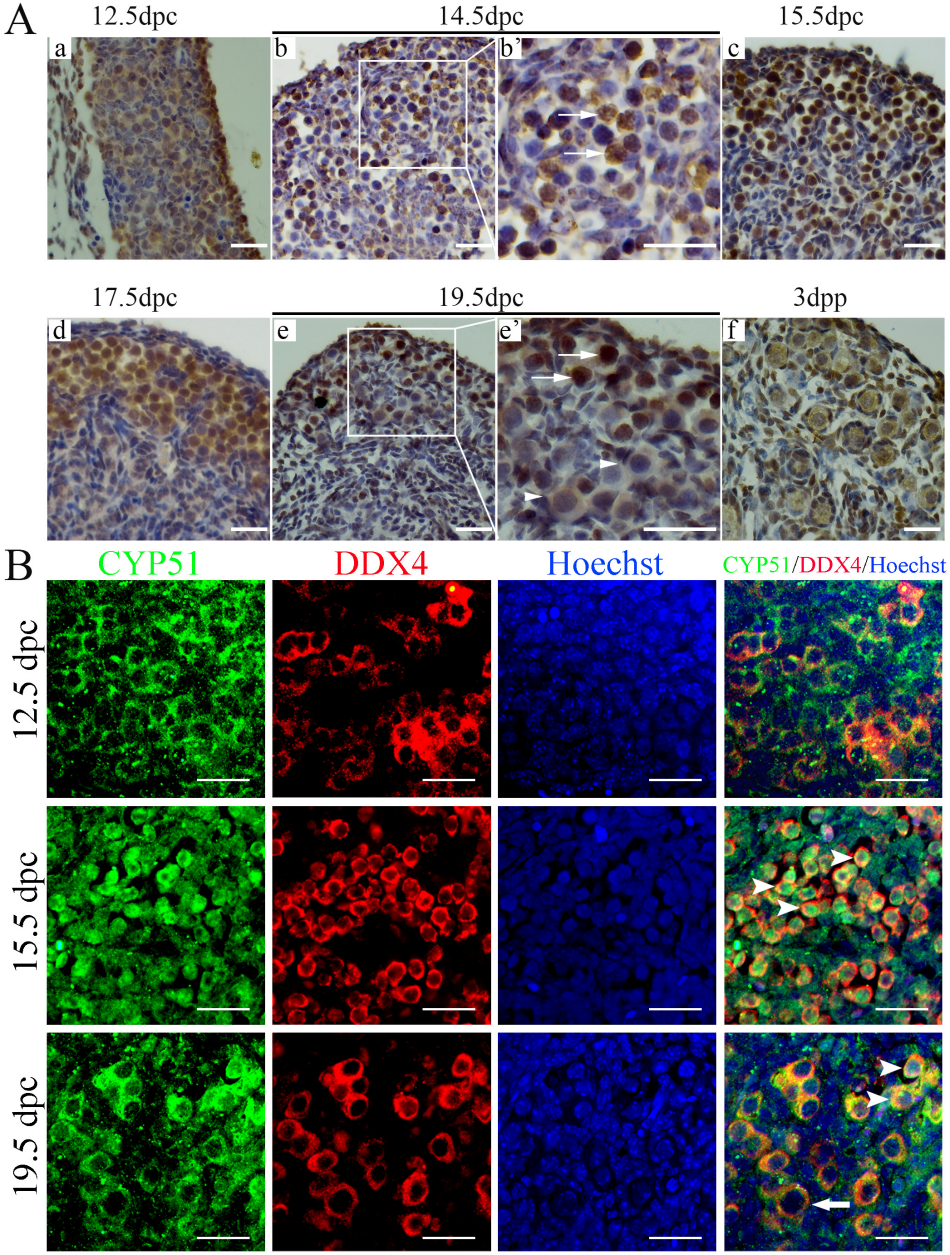
**Localization of CYP51 in developing mouse gonad.** (A) Immunohistochemical staining for CYP51 localization in mouse ovary. Mice ovaries from the 12.5 dpc (a), 14.5 dpc (b,b′), 15.5 dpc (c), 17.5 dpc (d), 19.5 dpc (e,e′) and 3 dpp (f) groups were immunostained for CYP51 (brown). CYP51 was primarily expressed in the cytoplasm of germ cells before 12.5 dpc and in the nucleus after 14.5 dpc, while the oocytes in the primordial follicles expressed weak nuclear CYP51. In the enlarged image (b′) of 14.5 dpc ovary, the arrows indicate germ cell nuclei with CYP51 staining. In the enlarged image (e′) of 19.5 dpc ovary, the arrows indicate oocytes in the cysts, while the arrowheads indicate primordial follicles. Scale bars: 25 μm. (B) Immunofluorescence staining for CYP51 localization in mouse ovary. Sections from 12.5 dpc, 15.5 dpc and 19.5 dpc mouse ovary were stained with antibodies against CYP51 (green) and DDX4 (red). Hoechst (blue) was used to mark cell nuclei. Arrowheads indicate nuclear CYP51 staining in germ cells; arrows indicate primordial follicle. Scale bars: 25 μm.

The spatiotemporal expression pattern of CYP51 in the fetal ovary was assessed by immunohistochemistry (Fig. 1A). Germ cells were recognized by their larger size and spherical nuclei. CYP51 staining was present in the cytoplasm of both somatic and germ cells in fetal ovaries as early as 12.5 dpc before meiosis initiation (Fig. 1Aa). At 14.5 dpc, when the germ cells entered meiosis, CYP51 became restricted to the nucleus in approximately 40% of the germ cells (arrows), while in the somatic cells around the cysts, CYP51 expression decreased (Fig. 1Ab,b′). By 15.5 dpc, when most of the germ cells had reached the zygotene stage, the germ cell nuclei in the cysts displayed strong staining for CYP51 (Fig. 1Ac). This strong nuclear-staining pattern in germ cells remained consistent until 17.5 dpc, when most of the oocytes were in the pachytene stage, while in the somatic cells surrounding the cysts, CYP51 displayed only background-level staining (Fig. 1Ad). With the achievement of the diplotene stage and the appearance of primordial follicles at 19.5 dpc, CYP51 expression in the nucleus was restricted to the oocytes within the cysts localized in the cortex of the ovary (arrows), while CYP51 was localized primarily in the oocyte cytoplasm, as well as in the pre-granulosa cells of the primordial follicles in the medulla (arrowheads) (Fig. 1Ae,e′). CYP51 expression pattern in the primordial follicles was more prominent at 3 days postpartum (dpp), when the primordial follicle pool in mice ovaries was established (Fig. 1Af).

Further, to confirm the specific location of CYP51 in mice fetal ovaries, we applied immunofluorescence with antibodies against both CYP51 (green) and DDX4 (DEAD box polypeptide 4) (red), which marked germ cells. Hoechst was used to mark cell nuclei (blue). As shown in Fig. 1B, CYP51 was primarily located in the cytoplasm of both the somatic cells and the germ cells in the 12.5 dpc ovaries. By 15.5 dpc, CYP51 was translocated into the nuclei of germ cells (arrowheads). In 19.5 dpc, when primordial follicles began to form, CYP51 displayed varied expression patterns. It was located either within the nucleus of germ cells in the cysts (arrowheads) or in the cytoplasm of the oocytes in the primordial follicles (arrow). In summary, CYP51 showed intense nuclear expression during the meiotic prophase I period in oocytes.

Meanwhile, we explored the location of CYP51 in prepubertal mouse testes when germ cells are undergoing the first wave of spermatogenesis (Scherthan et al., 1996). In 3 dpp testes, high CYP51 levels were noted in the interstitial tissue, but immunostaining was not detected in the germ cell cytoplasm or nucleus. In 5 dpp testes, when meiosis initiated, CYP51 began to localize in the nuclei of partial germ cells near luminal of the seminiferous tubules and in the interstitial tissue. In 8 dpp testes, as the first wave of germ cells entered meiosis, the nucleus of germ cells at the center of the seminiferous tubules began to stain for CYP51. Strong CYP51 staining in germ cell nucleus was constantly observed in the testes on 10 dpp and 12 dpp, when the spermatocytes reached the zygotene or pachytene stage, respectively. As in adult mice testes (2 months), CYP51 predominantly localized in cell nucleus of primary spermatocytes and elongating spermatids (Endo et al., 2015) (Fig. S1). In sum, the CYP51 expression in mice testes showed a similar pattern to that in ovaries, namely trans-location along with the meiotic progression.

### Cellular localization change of CYP51 in developing mouse ovaries

**Fig. 2.**
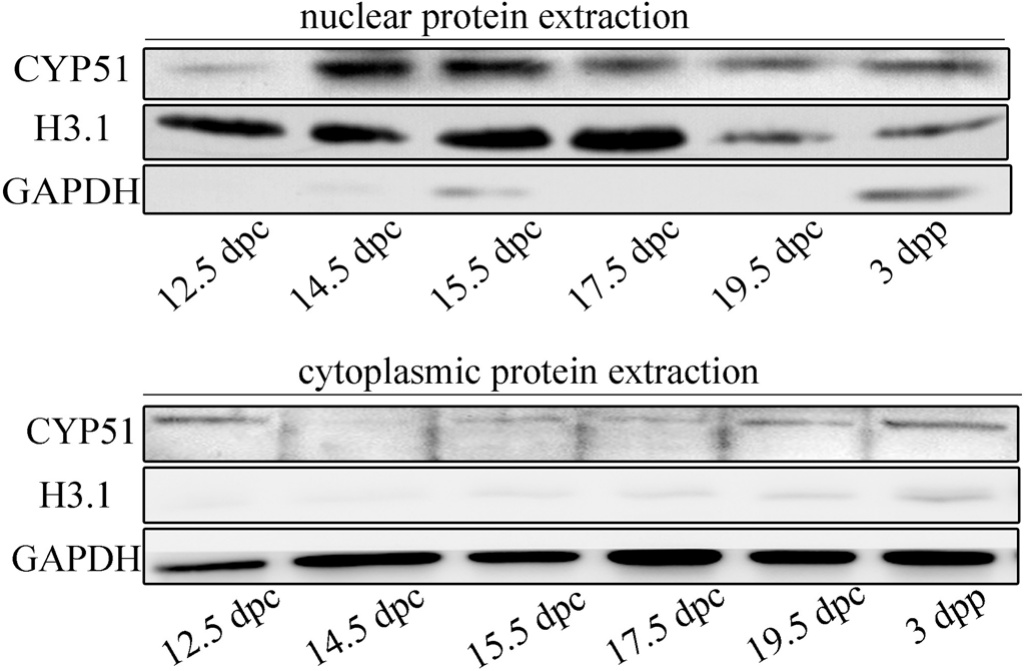
**Cellular localization change of CYP51 in developing mouse ovaries.** Western blotting analysis of CYP51 in nuclear/cytoplasmic extracts from mouse ovaries. H3.1 was used as nuclear protein reference and GAPDH was used as cytoplasmic protein reference.

The trans-locating localization of CYP51 in fetal oocytes was reconfirmed through western blotting assays using the nuclear protein extracted from fetal ovaries (Fig. 2). It showed that CYP51 protein level in the nuclear extraction was low at 12.5 dpc, but began to increase steadily from 14.5 dpc afterwards. On 19.5 dpc, the protein level of CYP51 decreased significantly. In contrast, CYP51 protein level in the cytoplasmic extraction was relatively high on 12.5 dpc, but kept at lower levels from 14.5 dpc afterwards. The protein level of CYP51 in cytoplasm rose up on 3 dpp. Histone H3.1 was used as a nuclear reference and GAPDH was used as a cytoplasmic reference. The localization pattern indicated a nuclear trans-location tendency of CYP51 in pace with meiotic prophase I progression, which was consistent with the immunostaining studies in fetal ovaries, implying a potential functional role of CYP51 in pace with meiotic prophase I progression.

### RA signaling regulates CYP51 distribution in the oocyte nucleus

RA is the key signal that induces meiosis in both sexes. As CYP51 was localized in the nucleus of germ cells undergoing meiotic prophase I, we examined whether RA signaling influenced CYP51 expression patterns. Germ cells usually responded to RA signaling and initiated meiosis at around 13.5 dpc in fetal ovary in mice (Edson et al., 2009). Therefore, 12.5 dpc ovaries were cultured *in vitro* with either exogenous RA for 1 day or RA receptor antagonist for 3 days before examining the activation or blockage effect of RA on CYP51 expression.

**Fig. 3.**
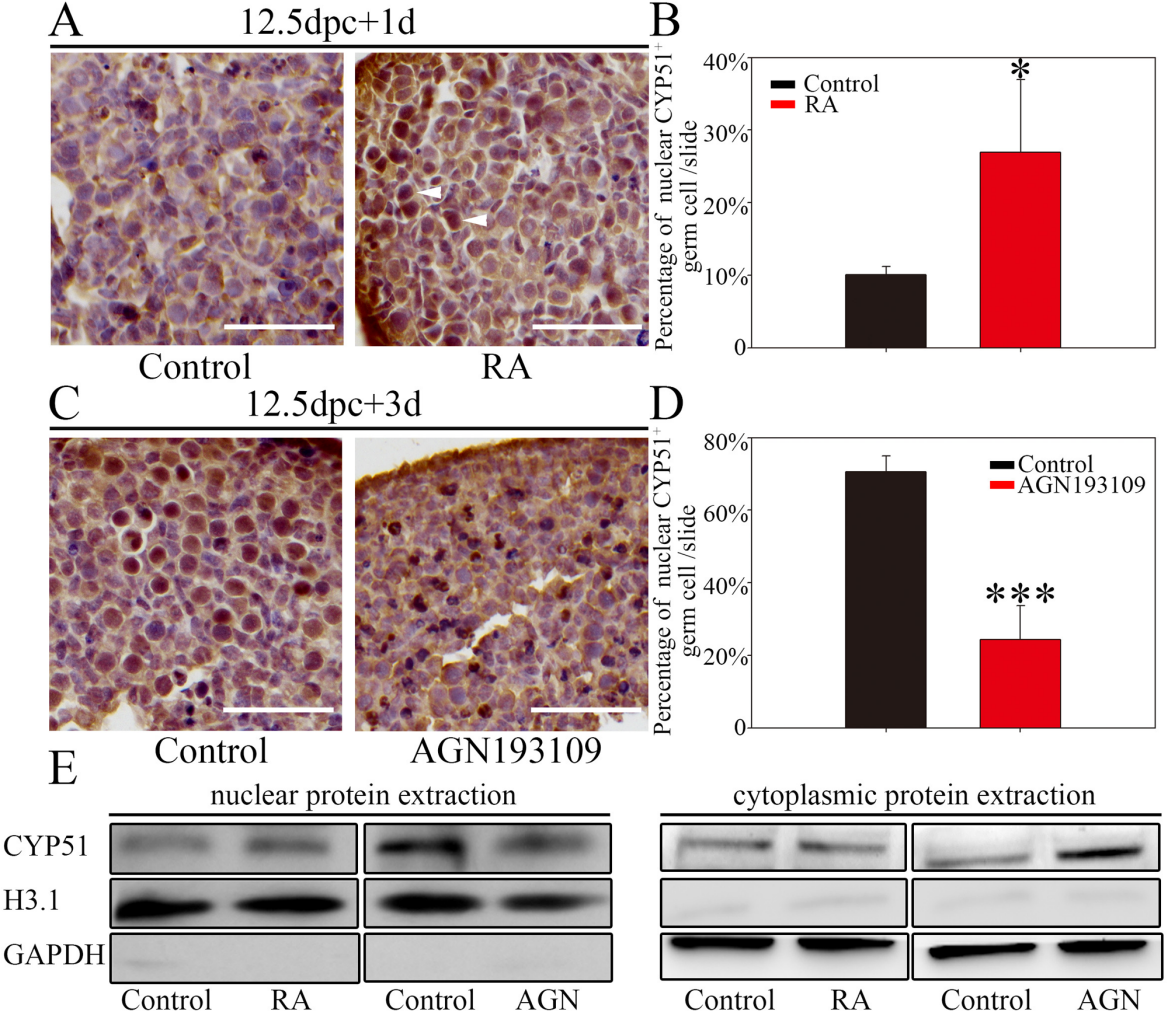
**RA regulates the shuttling of CYP51 from the cytoplasm to the nucleus in fetal mouse ovaries.** (A,B) RA treatment promoted nuclear CYP51 expression. Ovaries at 12.5 dpc were cultured with 1 μM RA for 1 day *in vitro*. (A) Histological sections were stained with an anti-CYP51 antibody. Arrowheads indicate germ cells with CYP51 nuclear staining. Scale bars: 25 μm. (B) Bar chart showing the percentage of nuclear CYP51-positive germ cells in total germ cells per slide. (C,D) RA receptor inhibition reduced nuclear CYP51 expression. Mice ovaries at 12.5 dpc were cultured with 5 μM AGN193109 for 3 days *in vitro*. (C) Histological sections were stained with an anti-CYP51 antibody. Scale bars: 25 μm. (D) Bar chart showing the percentage of nuclear CYP51-positive germ cells in total germ cells per slide. The data are presented as the means±s.e.m. of 3–9 ovaries per group. Asterisk (*) denotes a statistically significant difference between the control and the treatment groups. *P<0.05 and ***P<0.001 (*t*-test). (E) Western blotting analysis of CYP51 expression in nuclear/cytoplasmic extracts from 12.5 dpc ovaries treated with RA or AGN193109. H3.1 was used to normalize nuclear protein distribution, and GAPDH used as cytoplasmic protein reference.

When 12.5 dpc ovaries were cultured *in vitro* in the presence of 1 μM RA for 1 day (equaling 13.5 dpc), germ cells with CYP51 nuclear staining (arrowheads) were significantly more numerous than in the control (Fig. 3A). Quantification statistic showed that the percentage of germ cells with nuclear CYP51 staining in RA group was 2.7 times as many as those in the control (26.94%±9.98% for RA versus 10.09%±1.14% for control per slide) (*P*<0.05) (Fig. 3B). Conversely, when 12.5 dpc ovaries were exposed to RA receptor antagonist AGN193109 and cultured *in vitro* for 3 days (equaling 15.5 dpc), the percentage of germ cells with nuclear CYP51 staining was significantly reduced compared to the control (24.31%±9.34% for AGN193109 versus 70.63%±4.32% for control per slide) (*P*<0.001) (Fig. 3C,D).

Furthermore, we examined CYP51 levels in nuclear protein extracted from 12.5 dpc ovaries treated with RA or AGN193109. The western blotting results consistently showed that CYP51 levels in the nuclear protein fraction significantly increased after RA treatment for 1 day, but decreased after AGN193109 treatment for 3 days (Fig. 3E). Meanwhile, CYP51 levels in the cytoplasmic protein fraction did not show significant variation following RA treatment, but increased significantly following AGN193109 treatment, indicating that activation of RA signaling is positively related to the localization of CYP51 in fetal ovary (Fig. 3E).

We surmised that the premature nuclear distribution of CYP51 induced by exogenous RA might be associated with early meiotic stimulation in germ cells, while the disappearance of CYP51 nuclear localization caused by AGN193109 was due to the lack of meiotic stimulation. In summary, CYP51 nuclear localization coincides with germ cell entry into meiosis, and is regulated by RA signaling, suggesting that CYP51 may function during meiotic prophase I downstream of RA signaling.

### CYP51 participates in zygotene/pachytene transition in oocytes

**Fig. 4.**
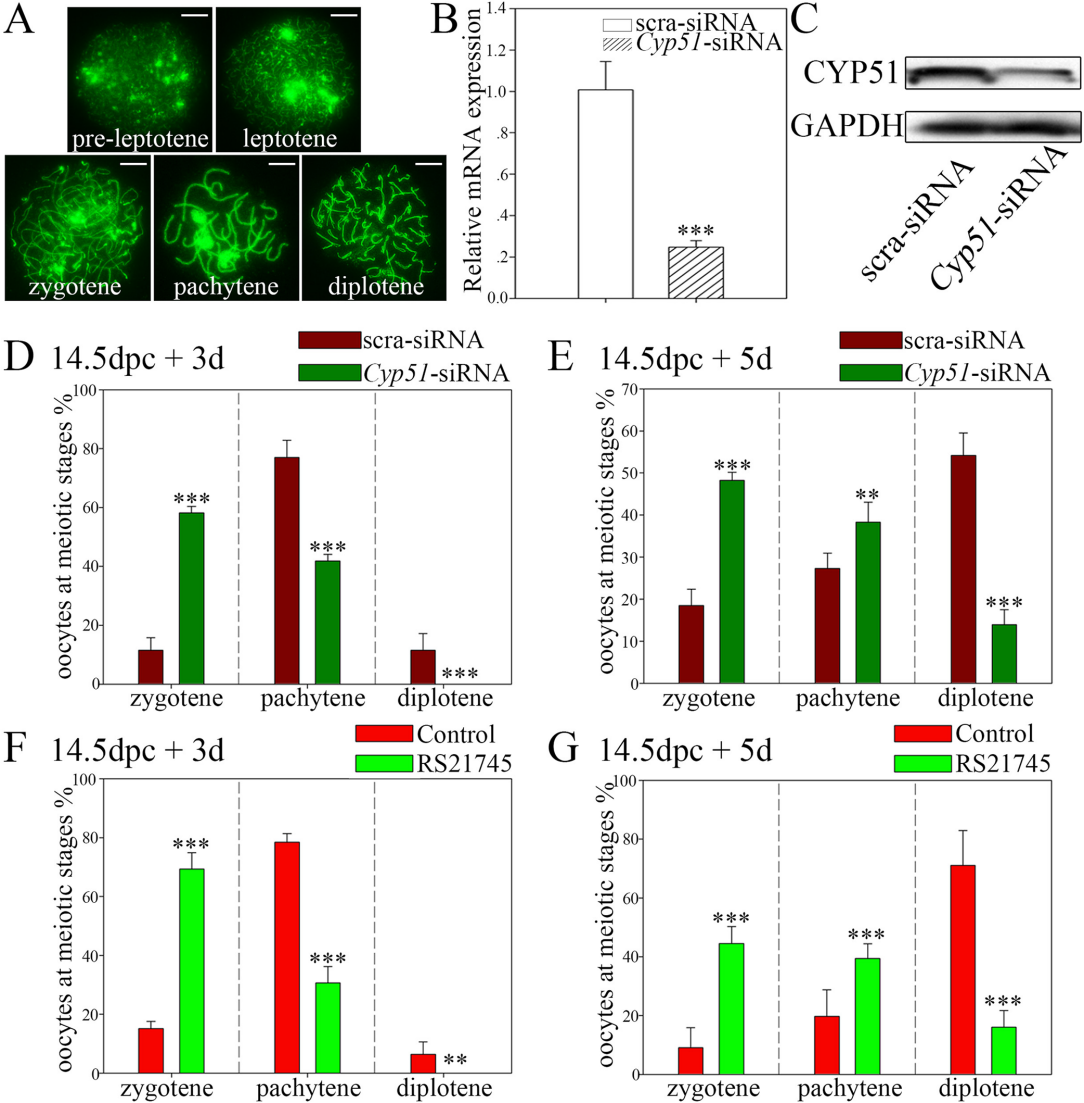
**Suppression of CYP51 impedes the zygotene/pachytene transition.** (A) Typical images of the substages of meiotic prophase I, including the pre-leptotene, leptotene, zygotene, pachytene and diplotene stages. Oocytes chromosomes were stained for SCP3 (green) to determine the specific substage. Scale bars: 10 μm. (B,C) Interference efficiency of *Cyp51*-siRNA in 14.5 dpc ovaries. (B) qRT-PCR analysis of *Cyp51* mRNA levels with scra-siRNA or *Cyp51*-siRNA treatment (normalized to *β-actin*). (C) Western blotting analysis of CYP51 protein level with scra-siRNA or *Cyp51*-siRNA treatment GAPDH was used as internal reference. (D,E) Knockdown of *Cyp51* by siRNA impedes meiotic prophase progression in oocytes. Statistical analysis of the quantification results from the chromosomal spread examination using 14.5 dpc ovaries cultured *in vitro* with *Cyp51*-siRNA treatment for 3 days (D) and 5 days (E). The meiotic progression of oocytes from *Cyp51*-siRNA treated ovaries was significantly delayed; fewer oocytes entered the pachytene stage than in the scra-siRNA group. (F,G) RS21745 treatment blocked meiotic prophase progression in oocytes. Statistical analysis of the quantification results from the chromosomal spread examination using 14.5 dpc ovaries cultured *in vitro* with RS21745 treatment for 3 days (F) and 5 days (G). The results reconfirmed previous data showing that inhibition of CYP51 impedes the zygotene-to-pachytene transition in oocytes. Unidentified germ cells were not included in the analyses. More than 300 oocytes were counted in each group. The data are presented as the means±s.e.m. of 3–9 ovaries per group. Asterisk (*) denotes a statistically significant difference between the control and the treatment groups. **P<0.01, and ***P<0.001 (*t*-test).

Oocyte chromosome spreading facilitates accurate analysis of the progression of meiotic prophase I. The stages of meiotic prophase I in individual oocytes were identified based on the appearance of the axial elements (AEs) (Fig. 4A). We also detected CYP51 localization in different substages of meiotic prophase I, and found that CYP51 displayed intensive localization in the nuclei of oocytes in the zygotene and pachytene stages (Fig. S2). This pattern suggested that CYP51 plays a role in the zygotene and pachytene stages.

Since most oocytes pass through the zygotene and the pachytene stages after 14.5 dpc, 14.5 dpc ovaries were collected and cultured *in vitro* in order to determine the function of CYP51 in meiotic prophase I. Using a small interference RNA (siRNA)-mediated gene knockdown approach, we isolated and injected 14.5 dpc mouse ovaries with siRNA and Trypan Blue mixture. The siRNA knockdown efficiency in mouse ovaries was examined by qRT-PCR and western blotting after 3 days in culture (Fig. 4B,C). The examination results revealed that CYP51 expression was reduced at both the mRNA and the protein level after siRNA treatment.

After 3 days *in vitro* culture (equaling 17.5 dpc), most of the oocytes in the scrambled siRNA group had reached the pachytene or diplotene stage; whereas oocytes in the *Cyp51*-siRNA group progressed through the leptotene stage, however, most were arrested at the zygotene stage, an effect accompanied by a significant reduction in the distribution of pachytene oocytes (*P*<0.001) (Fig. 4D). Similar results were observed after 5 days of *Cyp51*-siRNA treatment (equaling 19.5 dpc), as almost 50% of the oocytes in the knockdown group arrested at the zygotene stage, versus >70% of the oocytes in the control group had reached the diplotene stage (Fig. 4E).

Meanwhile, 14.5 dpc ovaries were treated with RS21745, which specifically inhibits CYP51. We observed that the nuclear staining of CYP51 was unaffected following RS21745 treatment (arrowheads) (Fig. S3A). The majority of the oocytes were arrested at the zygotene stage after 3 or 5 days treatment with RS21745 (*P*<0.001) (Fig. 4F,G), suggesting that CYP51, or some intermediate products in the cholesterol biosynthesis reaction catalyzed by CYP51, may participate in prophase I progression. Moreover, to explore whether CYP51 acted on meiotic progression through FF-MAS, which was the production of CYP51 enzymatic activity and induced resumption of meiosis in oocytes, we added FF-MAS in the presence of RS21745 to the cultured ovaries. The results showed that a supplement of FF-MAS could not restore the meiotic arrest of germ cells following CYP51 inhibition, as the majority of the oocytes from the FF-MAS supplementation groups were arrested at the zygotene stage, which displayed an insignificant difference from the CYP51 inhibition group (Fig. S3B).

Since most germ cells in the 14.5 dpc mice ovaries were in the pre-leptotene and leptotene stages (Fig. S3C) and could progress normally to the zygotene stage in the treated group, we postulated that CYP51 suppression did not affect early progression of meiotic prophase I. Additionally, to determine whether CYP51 is involved in meiotic prophase I after the pachytene stage, we treated 17.5 dpc ovaries with RS21745 for 2 days, because the majority of oocytes in 17.5 dpc ovaries went through the pachytene stage (Fig. S3D). We noted no significant difference in oocyte meiotic distribution between the control and treatment groups (Fig. S3E). Thus, we surmised that the function of CYP51 is restricted to the transition from the zygotene stage to the pachytene stage.

### CYP51 inhibition down-regulates the expression of the cohesin subunits REC8 and STAG3

**Fig. 5.**
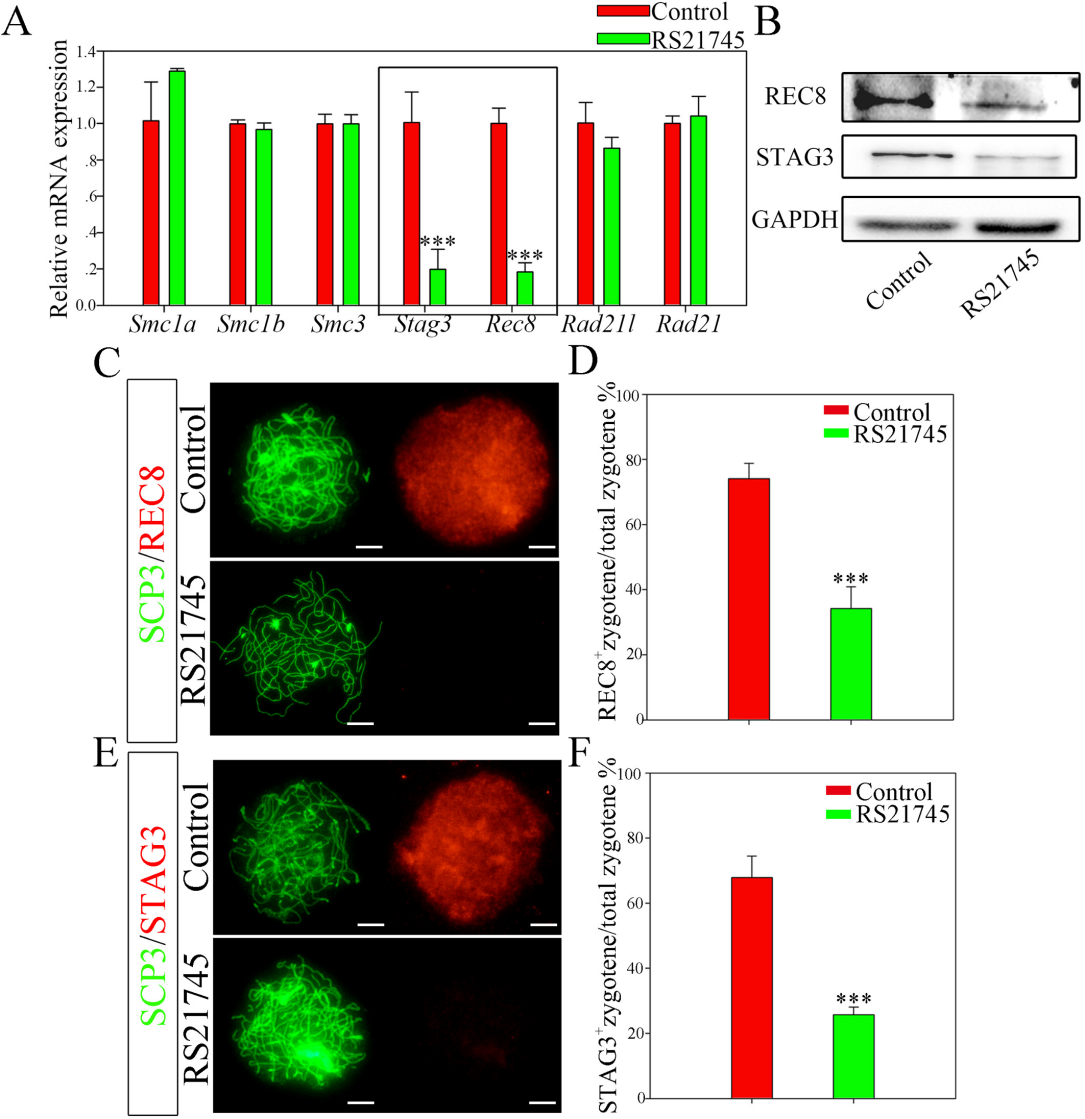
**CYP51 regulates the expression of the meiosis-specific cohesin subunits REC8 and STAG3.** (A,B) RS21745 treatment decreased REC8 and STAG3 expression. Ovaries at 14.5 dpc were cultured with 10 μM RS21745 for 3 days *in vitro*. (A) Expression of the cohesin subunits following RS21745 treatment by qRT-PCR. All qRT-PCR values were normalized to *β-actin* and were expressed as a relative ratio to the control; the means±s.e.m. of 3 values are shown. (B) Expression of REC8 and STAG3 following RS21745 treatment by western blotting GAPDH was used as internal reference. (C–F) Inhibition of CYP51 disturbed the distribution of both REC8 and STAG3 on the chromosomes. (C,E) In the control groups, REC8 and STAG3 expression was observed at high levels in zygotene oocytes. In the CYP51 inhibition groups, the cohesin signals were absent in zygotene cells. (D,F) The percentage of REC8-positive or STAG3-positive oocytes at the zygotene stage was recorded. Unidentified germ cells were not included in the analyses. Scale bars: 10 μm. The data are presented as the means±s.e.m. of 3–9 ovaries per group. Asterisk (*) denotes a statistically significant difference between the control and the treatment groups. ***P<0.001 (*t*-test).

Based on the fact that RS21745 treatment blocked oocytes from reaching the pachytene stage, when the paired homologous chromosomes establish synapses and strengthen their associations with the SC via cohesin, we next explored whether cohesin expression and localization on the SC was altered following CYP51 inhibition. qRT-PCR analysis of mRNA extracted from 14.5 dpc ovaries treated with RS21745 for 3 days showed that both *Rec8* and *Stag3* expression levels significantly decreased compared with the control group (*P*<0.001) (Fig. 5A), and western blotting confirmed that REC8 and STAG3 protein levels also decreased in RS21745-treated ovaries (Fig. 5B).

Chromosome spreading studies showed that both REC8 and STAG3 were restrictedly co-localized with the SC in zygotene oocytes (Fig. 5C,E). Strikingly, when we compared oocytes from the control and RS21745-treated groups, we found that significantly fewer zygotene oocytes in RS21745-treated ovaries than in control ovaries were positive for both cohesins (*P*<0.001), indicating that inhibition of CYP51 retarded REC8 and STAG3 expression in the zygotene SC (Fig. 5D,F). These results suggested that following CYP51 inhibition, oocytes were arrested at the zygotene stage, which might be correlated to the deficiency in the two cohesin subunits.

### Failure of entering the diplotene stage interrupts primordial follicle assembly

Previous studies have shown that the completion of meiotic prophase I and formation of primordial follicle are causally related (Paredes et al., 2005; Wang et al., 2015). As CYP51 suppression resulted in delayed meiotic progression, we wondered whether it also retarded germ cell cyst breakdown and primordial follicle formation. To study the action of CYP51 on germ cell cyst breakdown that occurs around birth (19.5 dpc) in mice ovaries, 14.5 dpc ovaries were cultured for 8 days (equaling 3 dpp, when primordial follicle formation completed) through either knockdown of *Cyp51* or inhibition of CYP51 activity.

**Fig. 6.**
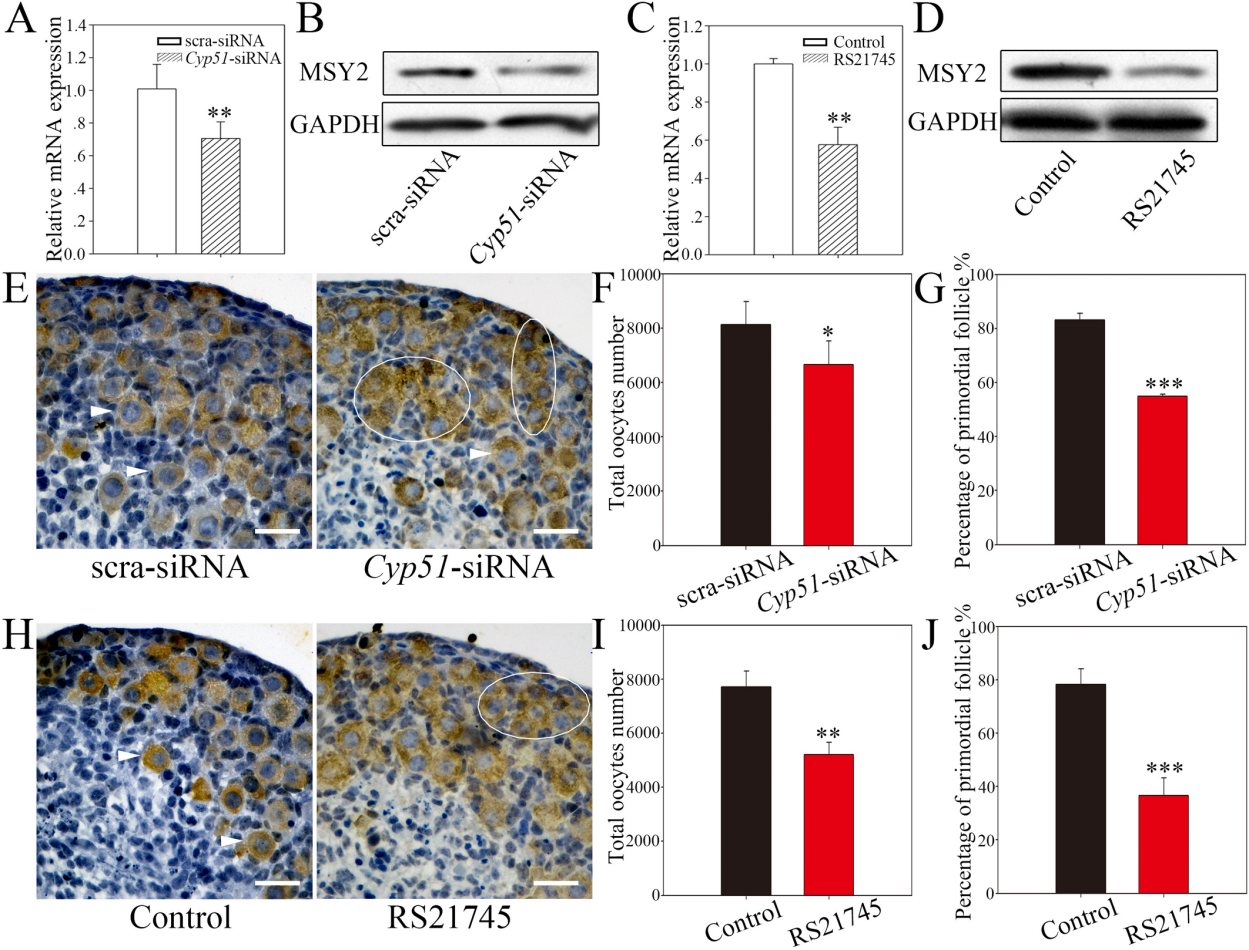
**Suppression of CYP51 inhibited cyst breakdown and primordial follicle formation.** (A–D) Suppression of CYP51 decreased MSY2 expression in ovaries. MSY2 was downregulated by siRNA-mediated knockdown of *Cyp51* (A,B) or inhibition of CYP51 (C,D), as determined by qRT-PCR and western blotting. All qRT-PCR values were normalized to *Ddx4* and were expressed as a relative ratio to the control; the means±s.e.m. of 3 values are shown. GAPDH was used as internal reference. (E–J) Suppression of CYP51 resulted in cyst breakdown failure and primordial follicle formation reduction. Ovaries at 14.5 dpc were cultured *in vitro* with (E–G) *Cyp51*-siRNA treatment or (H–J) in the presence of 10 μM RS21745 for the first 5 days and without the chemical for another 3 days. (E,H) All histological sections were stained with an anti-DDX4 antibody, which was used to identify the germ cells. The arrowheads indicate primordial follicles, and the white circles indicate germ cell cysts. (F,I) The total number of oocytes and (G,J) the percentage of primordial follicles were analyzed. Scale bars: 25 μm. The data are presented as the means±s.e.m. of 3–9 ovaries per group. Asterisk (*) denotes a statistically significant difference between the control and the treatment groups. *P<0.05, **P<0.01, and ***P<0.001 (*t*-test).

After 5 days of culture following *Cyp51*-siRNA injection or RS21745 treatment in 14.5 dpc ovaries (equaling 19.5 dpc), the expression of Y box protein 2 (MSY2), a molecular marker of diplotene, was significantly decreased at both the mRNA and the protein level (Fig. 6A–D), indicating that the oocytes were delayed in reaching the diplotene stage following CYP51 inhibition. After 8 days of culture (equaling 3 dpp), in scrambled siRNA-treated group, few germ cell cysts were observed and most oocytes were enclosed by pre-granulosa cells to form primordial follicles (83.1±2.6%), while in the *Cyp51*-siRNA groups more oocytes remained in the cysts and the formed primordial follicles were significantly fewer than in the scrambled siRNA-treated group (54.9±0.8%) (Fig. 6E–G). As CYP51 expression in the somatic cells began at 19.5 dpc, we treated 14.5 dpc ovaries with RS21745 for the first 5 days and then cultured them without the chemical for the following 3 days. Similarly, primordial follicle formation was significantly reduced in the CYP51 inhibiting group compared with the control group (*P*<0.001) (Fig. 6H–J). To sum up, we observed germ cell cyst breakdown failure and primordial follicle formation reduction following CYP51 block.

Moreover, we noticed that the oocyte population was smaller following both siRNA and RS21745 treatment (scrambled siRNA, 8121±844; *Cyp51*-siRNA, 6664±864; control, 7708±592; and RS21745, 5200±458) (Fig. 6F,I), which suggests that some oocytes that could not progress through the zygotene stage may have undergone apoptosis.

## DISCUSSION

The results of this study demonstrated that CYP51 displayed nuclear localization during meiotic prophase I in germ cells, and that CYP51 promoted zygotene/pachytene transition. Meanwhile, blockage of CYP51 is correlated to the decreased expression of meiosis-specific cohesion subunits REC8 and STAG3. Moreover, oocytes were retarded in reaching the diplotene stage and primordial follicle assembly was impeded following CYP51 blockage as well, which served as additional evidence indicating that only those oocytes that reached the diplotene stage can be enclosed by granulosa cells to form primordial follicles in mouse ovaries.

Generally, CYP51 localizes to the endoplasmic reticulum of nucleated cells and is the only P450 family member involved in a housekeeping metabolic pathway (Rozman et al., 1996). Here we used a primary antibody, which targeted Ser^414^ to Cys^431^ of CYP51, to detect the localization of CYP51 in mouse gonad. Our results demonstrated that CYP51 displayed nuclear staining in both fetal oocytes and prepubertal testes during meiotic prophase I period. Further studies were needed to reveal whether the nuclear CYP51 protein was a truncated form or not. In reproductive processes, CYP51 synthesizes signaling sterols that act in both sexes. In males, CYP51 expression is widely distributed in the acrosomal membranes of germ cells in mice, bulls and rams (Cotman et al., 2004). Germ cell-conditional deletion of *Cyp51* in mice did not affect male reproductive performance. *Cyp51-*deleted testes were morphologically normal (Keber et al., 2013). In females, CYP51 is expressed spatiotemporally in mice ovaries, exhibiting cell-type-specific expression during meiotic resumption in oocytes (Rozman et al., 2002; Song et al., 2008). In our previous study, CYP51 expression in the fetal ovary is high between 13.5 dpc and 17.5 dpc (Zhang et al., 2009). This study demonstrated that CYP51 displayed similar nuclear translocation in both fetal ovaries and prepubertal testes during meiotic prophase I period. However, blockage of CYP51 disturbed meiotic progression in oocytes and resulted in reduced primordial follicle formation in fetal ovaries, which suggested a potential regulating role of CYP51 in female meiosis.

The results of this study implied that CYP51 is involved in RA induced oocytes meiosis. Previously, we and others demonstrated that RA, derived from the somatic environment of either the mesonephroi or the fetal ovary, is the key signal for the induction of meiosis initiation and progression in mice, as well as in humans (Bowles et al., 2006; Koubova et al., 2006; Mu et al., 2013). In this study, we proved that RA signaling regulates the nuclear distribution of CYP51 during meiotic prophase I in fetal mice ovary. Although it is unclear whether RA directly induced CYP51 translocation or CYP51 autogenously began to localize in the nucleus during meiotic progression, our observations indicate that the nuclear distribution of CYP51 is causally related to meiotic progression of germ cells under the regulation of RA. Additional evidence implying that CYP51 is involved in RA induced meiosis is that suppression of CYP51 decreased both REC8 and STAG3 expression, which are major targets of RA signaling in regulating cohesin subunits behaviors (Koubova et al., 2014). Further research is needed to explore the detailed mechanism of how RA induces the translocation of CYP51.

According to our results, CYP51 may be crucial for the zygotene/pachytene transition. CYP51 was notably accumulated in the nucleus of oocytes from 15.5 dpc to 17.5 dpc, when the majority of the oocytes were shifting from the zygotene stage into the pachytene stage. Considering that homologous chromosome synapses are established during the pachytene stage (Borum, 1961; Vazquez-Nin and Echeverria, 1976), we speculated that CYP51 is required for the synapse chromosomal events. It is known that at least three different cohesin complexes consisting of seven cohesin subunits functioned during meiotic prophase I (Lee and Hirano, 2011). Both REC8 and STAG3 are meiotic specific cohesins (Lee and Hirano, 2011; Prieto et al., 2001), which are regulated by RA signaling during the chromosomal program of meiotic prophase (Soh et al., 2015). In this study, we found that suppression of CYP51 decreased both REC8 and STAG3 expressions. Since RA directly regulates REC8 expression independent of *Stra8* (Koubova et al., 2014), it is reasonable to assume that RA induces CYP51 nuclear distribution and may act on regulating cohesin subunit expression during the pachytene stage. The question is how CYP51 regulates REC8 and STAG3 expression. According to our study, the distinct CYP51 localization and shuttling between the cytoplasm and nucleus that occurs in oocytes at different stages of meiosis suggests that CYP51 may act directly as a transcriptional factor or transcriptional co-regulator on the gene expression of cohesin subunits. However, we cannot exclude the possibility that being one of the key enzymes in cholesterol synthesis, CYP51 may also act indirectly through the sterol-mediated pathway on the gene expression of cohesin subunits. Thus, the mechanisms that regulate the nuclear import and export of CYP51 and its specific target remain to be elucidated.

In female mammals, the primordial follicle pool serves as a resting population of oocytes that are available during the reproductive life span. Although several factors, such as estrogen and progesterone, as well as the Jagged1-Notch signaling pathway, have been implicated in the establishment of primordial follicles (Chen et al., 2007; Trombly et al., 2009), less is known regarding the relationship between oocyte meiotic progression and primordial follicle formation, which occur within a narrow timeframe in the mouse ovary. According to a study by Paredes, premature loss of synaptonemal complex protein-1 (SCP1), which keeps homologous chromosomes together during the pachytene stage and dissociates during chromosomal desynapsis in the diplotene stage, resulted in the earlier arrival of oocytes to the diplotene stage, and increased the numbers of newly formed follicles (Paredes et al., 2005), suggesting that completion of meiotic prophase I endows oocytes with the ability to orchestrate follicle assembly. Our group demonstrated that meiotic progression of oocytes is closely correlated with increased levels of intra-oocyte cyclic AMP (cAMP), and reduction of cAMP levels inhibits primordial folliculogenesis in the perinatal mouse ovary (Wang et al., 2015). Additionally, our present and previous studies have shown that suppression of CYP51 in fetal mouse ovary blocks primordial folliculogenesis (Zhang et al., 2009). In this study, both *Cyp51*-siRNA and RS21745 treatment delayed oocyte entry into the diplotene stage, which resulted in cyst-breakdown inhibition and primordial follicle formation failure. These results are indicative of the existence of a relationship between oocytes reaching the diplotene stage and primordial follicle assembly, suggesting that the mutual communication between oocyte and somatic cells orchestrating folliculogenesis is not activated until the oocytes enter the diplotene stage. However, the potential relationship between increased cAMP expression and CYP51 expression in oocytes before primordial follicle formation requires further exploration.

In conclusion, this study provided evidence that the dynamic expression of CYP51 in germ cells may be strongly correlated with the progression of meiotic prophase I. CYP51 suppression decreases REC8 and STAG3 expression, arrests oocytes in the zygotene stage and reduces the number of formed primordial follicles.

## MATERIALS AND METHODS

### Animals

CD1 mice were purchased from the Laboratory Animal Center of the Institute of Genetics (Beijing, China), housed in a temperature- and light-controlled room and allowed free access to water and food. 6–8-week-old female mice were mated with fertile males overnight, and at noon on the following day (when the presence of a vaginal plug was confirmed), the pregnancy was designated as 0.5 dpc. All experiments were performed in accordance with institutional and national guidelines and regulations and were approved by the China Agricultural University Animal Care and Use Committee.

### Culture media and chemicals

Serum-free Dulbecco's modified Eagle's medium/Ham's F12 nutrient mixture (DMEM/F12, Gibco BRL, Carlsbad, USA) supplemented with 10 IU/ml penicillin-streptomycin and HEPES was used for *in vitro* fetal ovary culture. The media were equilibrated in a 37°C incubator with 5% CO_2_ for 2 h prior to use. All-*trans-*RA (Sigma Chemical Co., St. Louis, USA) and AGN193109 (Toronto Research Chemicals, Toronto, Canada) were first dissolved in Dimethyl Sulphoxide (DMSO); RS21745 and FF-MAS (Toronto Research Chemicals) were first dissolved in ethanol. The stock solutions were kept at −20°C, re-dissolved in DMEM/F12, and then added to the media before use. The final concentrations of RA, AGN193109 and RS21745 were 1 μM, 5 μM and 10 μM, respectively (Bowles et al., 2006; Zhang et al., 2009). Control media were supplemented with the same volume of DMSO or ethanol.

### Ovary dissection and *in vitro* culture

Ovaries were collected on the desired dpc and dissected carefully from the mesonephros in pre-chilled phosphate-buffered saline (PBS), as previously reported (Wen et al., 2009). The isolated ovaries were subsequently transferred to and immersed in 1 ml of culture media in individual wells of a 6-well plate (NEST Biotechnology, Wuxi, Jiangsu, China) and incubated in a 37°C incubator with 5% CO_2_. One ovary per pair was used in the control group and the other was used in the treatment group.

### Histological sections and immunohistochemistry

Collected ovaries or testes were fixed in 4% paraformaldehyde (PFA) in PBS overnight and then transferred to 70% ethyl alcohol, dehydrated and embedded in paraffin, and then cut into 5-μm-thick sections. After being deparaffinized, rehydrated and subjected to antigen retrieval using 0.01% sodium citrate buffer (pH=6.0), the sections were immunostained overnight at 4°C with the following primary antibodies and dilutions: MSY2 (1:200; #sc-21316, Santa Cruz Biotechnology), DDX4 (1:100; #ab27591, Abcam), and CYP51 (1:200; made by MBL Beijing Biotech, Beijing, China; antigen peptide: SWAERLDFNPDRYLQDNC, targeting 414-431 of CYP51 protein). ELISA and western blot testing of the antibody were provided in Fig. S4. The slides were then incubated with a biotinylated secondary antibody (Zhongshan, Beijing, China) and avidinbiotin-peroxidase (Zhongshan, Beijing, China). After 1 min of exposure to diaminobenzidine (Zhongshan, Beijing, China), the sections were counterstained with hematoxylin.

### Meiotic spread preparations and immunofluorescence staining

Synaptonemal complex protein 3 (SCP3) is present on the AEs of the synaptonemal complexes between homologous chromosomes during all meiotic prophase I stages (Moens and Spyropoulos, 1995). The SCP3 antibody recognizes short segments of chromosomal core elements at the pre-leptotene stage and fully formed elements at the pachytene stage (Tarsounas and Moens, 2001). Briefly, dispersed ovarian cells were treated with 1% trisodium citrate dehydrate solution for 20 min at room temperature. Chromosomal spreads with the same volume of 1% PFA containing 2% Triton X-100 were fixed for 6 h at room temperature before being blocked with ADB for 30 min (antibody dilution buffer containing 1% serum, 3‰ BSA and 1‰ Triton X-100) and incubated with the appropriate primary antibodies overnight at 37°C. SCP3 antibody (diluted 1:50; #sc-20845, Santa Cruz Biotechnology) was used for prophase stage detection; SCP3 antibody (diluted 1:100; #NB300-232, NOVUS Biologicals, USA) and REC8 antibody (1:50; #sc-15152, Santa Cruz Biotechnology) or STAG3 antibody (1:50; #sc-20341, Santa Cruz Biotechnology) were applied simultaneously for REC8 and STAG3 detection, respectively. The samples were incubated with a secondary antibody (TRITC-conjugated donkey anti-goat antibody and FITC-conjugated donkey anti-rabbit antibody, Santa Cruz Biotechnology) for 90 min at 37°C at a dilution of 1:50 in ADB. Finally, 20 μl of Vectashield mounting media (Applygen Technologies Inc., Beijing, China) was applied to each slide, after which a cover slip was applied and sealed in place.

All slides were directly viewed using a fluorescence microscope (Eclipse, Nikon, Japan) and the meiotic prophase I stages were identified based on the appearance of the AEs. The average percentages of germ cells in individual meiotic prophase stages were determined from three slides, each of which included at least three ovaries. Germ cells were randomly dispersed on the slides, and at least 300 germ cells were analyzed per slide. Unidentified germ cells were not included in the analysis.

### RNA extraction and real-time qRT-PCR

Total RNA from 10 ovaries was extracted using TRIzol reagent (Invitrogen), according to the manufacturer's protocol, washed in 75% ethanol and then dissolved in water. First-strand cDNA was synthesized by reverse transcription (Promega Reverse Transcription System) from 1 µg of total RNA. Gene expression changes were evaluated using real-time qRT-PCR for *Ddx4* and *Msy2* (markers for germline cell and diplotene); *Smc1α*, *Smc1β*, *Smc3*, *Stag3*, *Rec8*, *Rad21*, and *Rad21l* (cohesin subunits function in meiotic prophase I); and *Cyp51*. *Ddx4* was used to normalize germ cell numbers due to its expression in germ cells at all stages, irrespective of the mitotic or meiotic stage (Toyooka et al., 2000). For each sample, data were first normalized to *β-actin*, and then the expression of each gene was divided by expression of *Ddx4* respectively*.* This comparison allowed the calculation of levels of germ cell specific genes relative to the number of germ cells present in the tissue (Bowles et al., 2006; Guerquin et al., 2010). *β-actin* was used for normalization for analysis of *Cyp51* levels.

### Western blotting analyses

Western blotting analyses were conducted as previously described (Su et al., 2001). Briefly, total protein was extracted in MEM-R, according to the manufacturer's protocol (Pierce, Rockford, USA). The nuclear and cytoplasmic proteins from fetal ovaries were extracted using a Nuclear-Cytosol Extraction Kit (Applygen Technologies Inc., Beijing, China). Protein concentrations were measured by a BCA assay procedure (CellChip Beijing Biotechnology Company, Beijing, China). The samples were separated on 15% SDS–PAGE and then transferred to Protran nitrocellulose membranes (Schleicher & Schuell, Dassel, Germany), which were incubated with appropriate primary antibodies overnight at 4°C. Antibody against CYP51, H3.1 (#21137-1, Signalway Antibody, USA), REC8 (#ab192241, Abcam), STAG3 and MSY2 were used at dilutions of 1:500, 1:100, 1:500, 1:400 and 1:500, respectively. The appropriate peroxidase-conjugated secondary antibodies (#ZB2301, ZSGB-BIO, Beijing, China) were diluted 1:5000 in TBST. The membranes were visualized using an enhanced chemiluminescence detection system (Amersham, Arlington Heights, USA). GAPDH was used as an internal control for total protein detection, while H3.1 was used as an internal control to normalize nuclear protein expression.

### siRNA knockdown

We isolated and injected 14.5 dpc ovaries with 0.5 μl of scrambled siRNA or *Cyp51*-siRNA using a glass pipette under a stereoscopic microscope. These ovaries were then transferred to an electric transfection apparatus (ECM2001, BTX, USA), and treated with 3 times quasi-square pulses at a pulse strength of up to 40 V/cm. Interference efficiency was examined 48 h after transfection. *Cyp51*-siRNA was mix of 5′-GCGTAAAGATATGAAAGGATA-3′ and 5′-TATCCTTTCATATCTTTACGC-3′. The scrambled-siRNA sequence was 5′-ACGTGACACGTTCGGAGAATT-3′, which has no homology with any known mouse mRNA.

### Germ cell and primordial follicle quantification

Germ cell numbers were determined using a previously described approach (Wen et al., 2009). Briefly, 5 μm serial sections stained with MSY2 antibody and hematoxylin were ordered sequentially on the slides. Every fifth section was analyzed, and the cumulative germ cell and primordial follicle counts were multiplied by five because four-fifths of the ovary was not analyzed (Flaws et al., 2001).

### Statistical analyses

The data are reported as the mean±s.e.m. of experiments performed in triplicate. 3–9 ovaries per group were used for the chromosome spreading experiment, as well as the germ cell and primordial follicle quantification experiment. The distributions of oocytes at different stages of meiotic prophase in fetal ovaries were analyzed by ordinal regression after logit transformation of the data and by ANOVA using Sigmaplot version 9.01 software (SISTAT Software, Inc., Chicago, USA). Different letters indicated significant differences (*P*<0.05). Unstaged oocytes were omitted from the analyses. The quantification results and real-time qRT-PCR data were analyzed by *t*-tests using Sigmaplot version 9.01 software.

## Supplementary Material

Supplementary information
