# Supplementary material for: Retinoic acid-induced CYP51 nuclear translocation promotes meiosis prophase I process and is correlated to the expression of REC8 and STAG3 in mice
Source: Biol Open. 2018 Nov 15;7(11):bio035626. doi: 10.1242/bio.035626 (PMC6262859; doi:10.1242/bio.035626)
Supplement: Supplementary information [file biolopen-7-035626-s1.pdf]

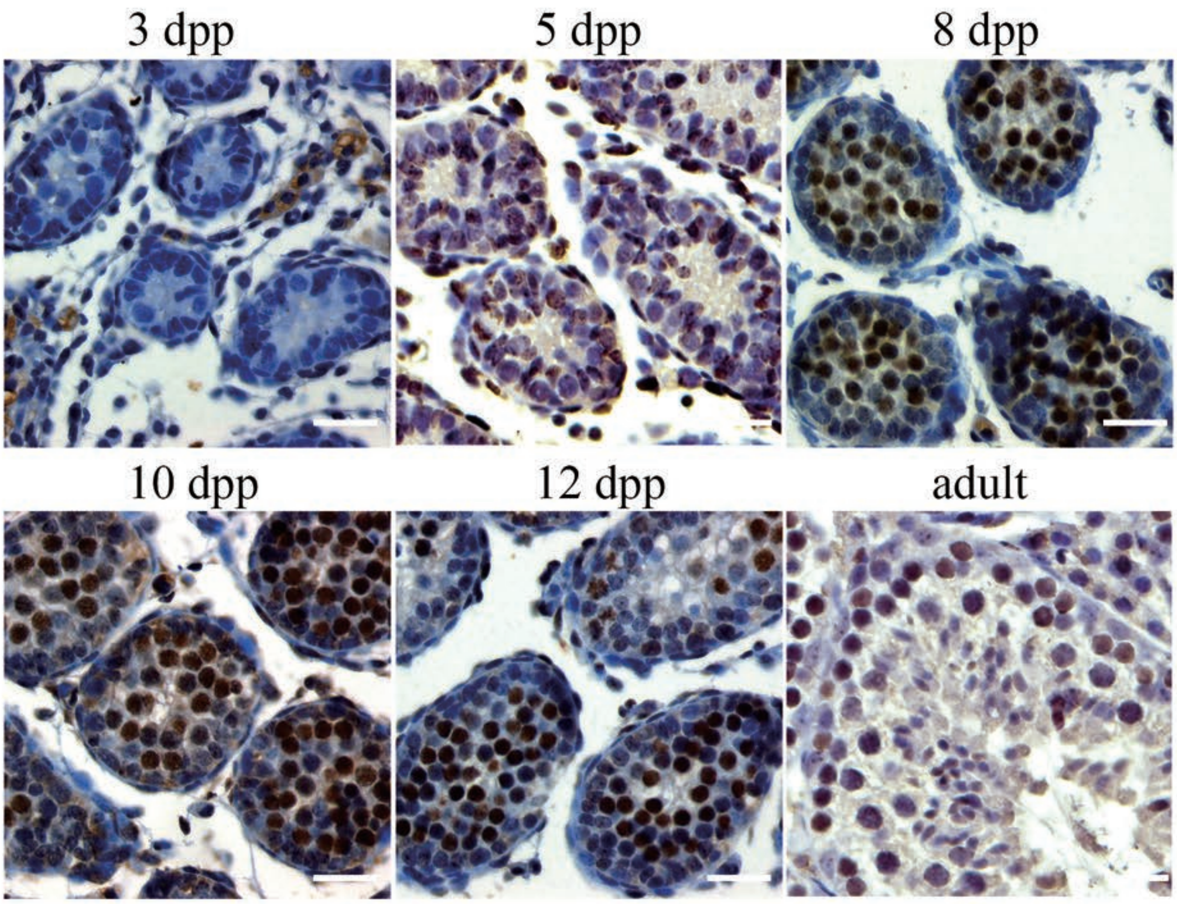

Figure S1. CYP51 expression in mouse testes. CYP51 was expressed in the interstitial tissue, but not in the germ cells of the 3 dpp testes. CYP51 immunostaining was obvious in germ cell nucleus at the center of the seminiferous tubules in 5 dpp, 8 dpp, 10 dpp and 12 dpp testes. In adult testes, CYP51 was predominantly expressed in nucleus of primary spermatocytes. Scale bar: 25 μm.

Biology Open • Supplementary information

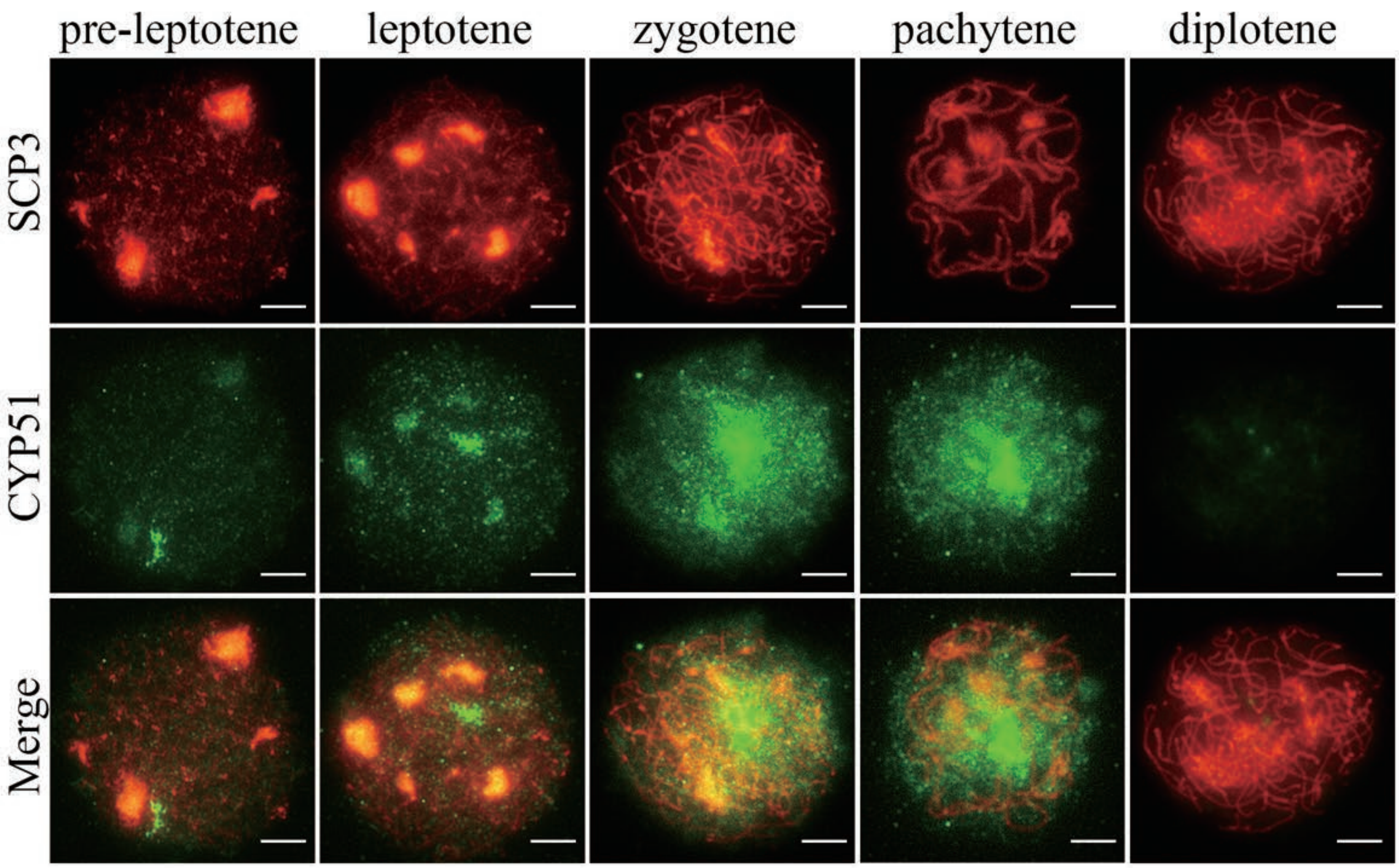

Figure S2. CYP51 expression in different substages of meiotic prophase I. Co-localization immunofluorescence staining for SCP3 (red) and CYP51 (green). Specific stages are determined according to the appearance of AEs. The expression of CYP51 was stronger in the zygotene and pachytene stage than other stages in oocytes. Scale bar: 10 µm.

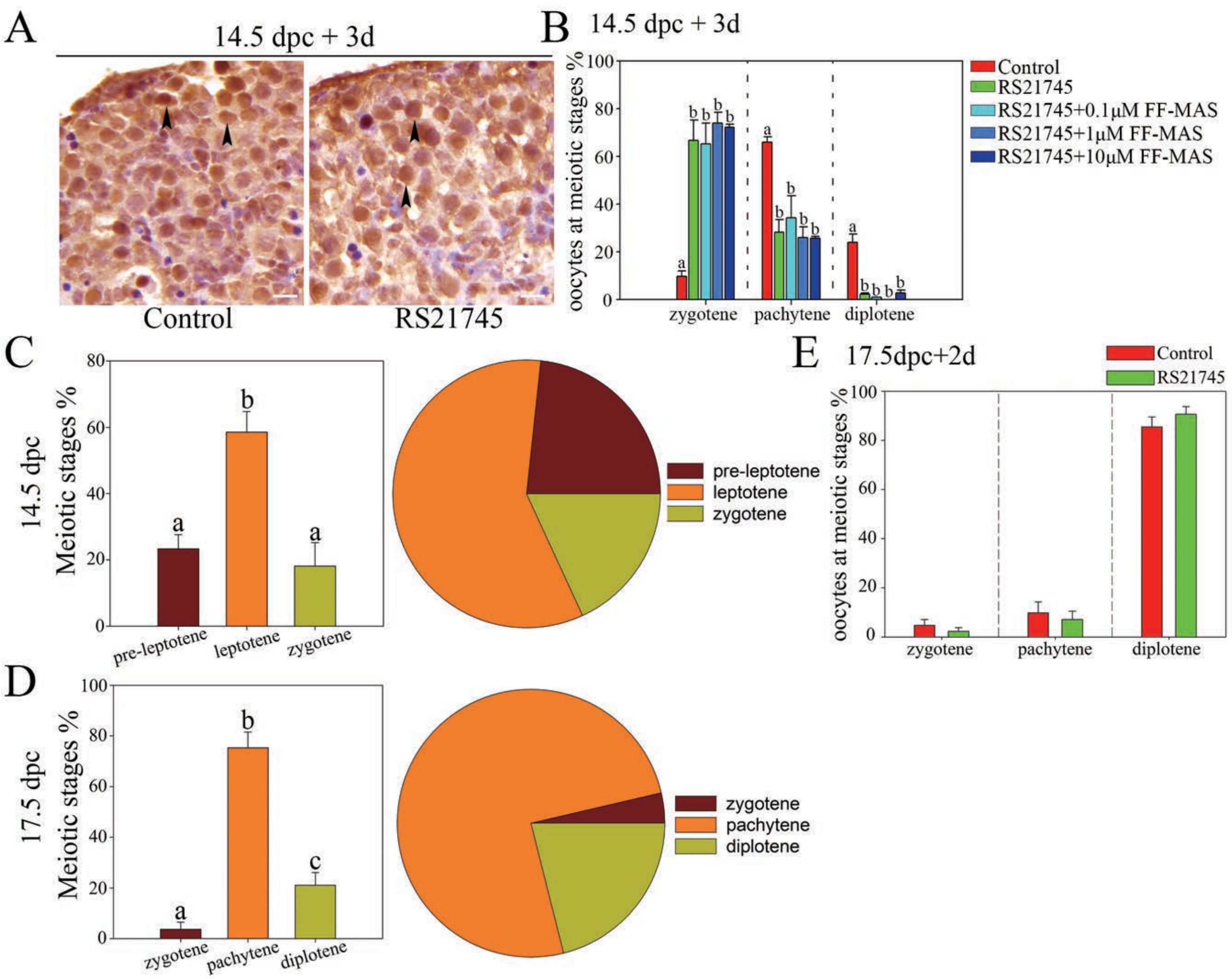

Figure S3. Supplementation of FF-MAS could not rescue the meiotic arrest following CYP51 inhibition. (A) The nuclear staining of CYP51 was not affected following RS21745 treatment. Ovaries at 14.5 dpc were cultured with RS21745 for 3 day *in vitro*. Histological sections were stained with an anti-CYP51 antibody. Arrowheads indicate germ cells with CYP51 nuclear staining. Scale bar: 25 μm. **(B)** FF-MAS cannot rescue the meiotic progression following CYP51 inhibition. Statistical analysis of the quantification results from the chromosomal spread examination using 14.5 dpc ovaries cultured *in vitro* with RS21745,

Biology Open • Supplementary information

RS21745 with FF-MAS (0.1µM, 1µM and 10 µM respectively) treatment for 3 days. **(C)(D)** Statistical analysis of the quantification results of meiotic substages of oocytes in **(C)** 14.5 dpc ovaries and **(D)** 17.5 dpc ovaries *in vivo*. **(E)** RS21745 treatment did not affect meiotic progression after the pachytene stage. Statistical analysis of the quantification results from the chromosomal spread examination using 17.5 dpc ovaries cultured *in vitro* with RS21745 treatment for 2 days. Unidentified germ cells were not included in the analyses. More than 300 oocytes were counted in each group. The data are presented as the means ± s.e.m. of 3-9 ovaries per group. Different letters (a-c) denotes a statistically significant difference between groups (ANOVA).

Biology Open • Supplementary information

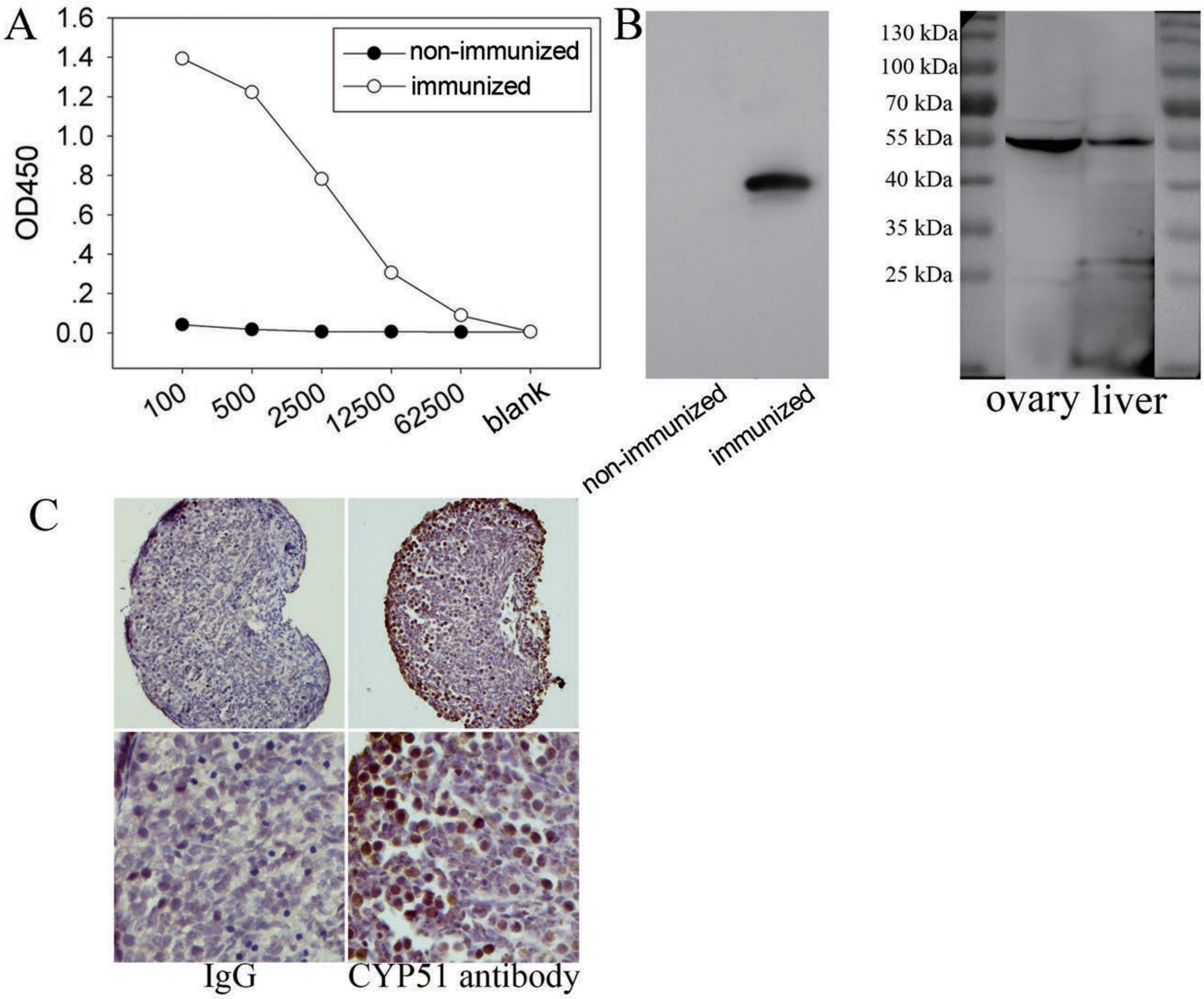

Figure S4. The CYP51 antibody production examination. (A) ELISA for antibody detection. **(B)** Whole Western blotting for antibody detection. Protein samples from adult mouse ovary and liver were used in the whole Western blot. CYP51 antibody production showed a single and clear band at ~55 kDa. **(C)** Left column: Negative control immunostaining section with rabbit IgG. Right column: Immunostaining section with CYP51 antibody production.
